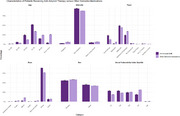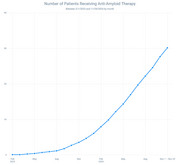# Characteristics and Safety Outcomes of Patients Treated with Lecanemab or Donanemab in Routine Clinical Practice: A Nationwide Study

**DOI:** 10.1002/alz70860_098805

**Published:** 2025-12-23

**Authors:** Jay B Lusk, Kim G Johnson, Andy Liu, Brian C. Mac Grory, Fan Li, Heather Whitson, Richard J O'Brien, Shannon Aymes, Bradley G Hammill, Emily C O'Brien

**Affiliations:** ^1^ University of North Carolina‐ Chapel Hill, Chapel Hill, NC, USA; ^2^ Duke University, Durham, NC, USA; ^3^ Duke University School of Medicine, Durham, NC, USA

## Abstract

**Background:**

Anti‐amyloid monoclonal antibodies (lecanemab and donanemab) received full approval by the FDA to treat early Alzheimer's disease and entered routine clinical practice in July 2023 and July 2024 respectively. Uptake patterns and post‐approval safety have not been fully characterized.

**Method:**

We used Epic Cosmos, a dataset of Epic health systems representing more than 289 million patients, to perform a retrospective study of patients who received donanemab or lecanemab infusions from July 1, 2023 to November 29, 2024. We compared patient demographics with patients with dementia who took oral donepezil, rivastigmine, galantamine, or memantine. We also reported cardiovascular risk factors and outcomes (MACCE, defined as stroke, myocardial infarction, or transient ischemic attack) among patients taking anti‐amyloid therapy.

**Result:**

A total of 3,402 patients received anti‐amyloid therapy, of whom 3,242 received lecanemab and 160 received donanemab. In the comparator group, 1,907,655 patients took oral dementia medications. The distribution of patients by age, race, ethnicity, primary insurance type, and ZIP code social vulnerability index quartile are shown in Figure 1. Patients receiving anti‐amyloid therapy were younger, more likely to be White and non‐Hispanic, more likely to have commercial insurance or Medicare and less likely to have Medicaid, more likely to be male, and were more likely to reside in a socioeconomically advantaged ZIP code. The number of patients receiving anti‐amyloid therapy increased rapidly from August 2023 (<250 patients) to November 2024 (>3,000 patients, Figure 2). Among patients receiving anti‐amyloid therapy, the average 10‐year ASCVD risk score was 19.9, 36.9% of patients had an ASCVD risk score of >10 within 12 months, and 84.6% of patients had a systolic blood pressure above 135 mmHg in the year prior to therapy; 1,345 (37.5%) of patients had a history of MACCE before therapy. Within 12 months of therapy initiation, 95 patients (2.7%, 95% CI 2.1‐3.2%) had a new MACCE diagnosis and 58 patients (1.7%, 95% CI 1.3‐2.1%) had a new diagnosis of a cerebrovascular event.

**Conclusion:**

Early adopters of anti‐amyloid therapy are, on average, more socially advantaged than the general population affected by dementia, although they exhibit high cardiovascular risk and high cardiovascular complication rates.